# Interleukin-22 Might Act as a Double-Edged Sword in Type 2 Diabetes and Coronary Artery Disease

**DOI:** 10.1155/2016/8254797

**Published:** 2016-10-18

**Authors:** Fangchen Gong, Jin Wu, Ping Zhou, Mengyao Zhang, Jingning Liu, Ying Liu, Xiang Lu, Zhengxia Liu

**Affiliations:** ^1^Department of Geriatrics, The Second Affiliated Hospital, Nanjing Medical University, Jiangsu 210029, China; ^2^Department of Neurology, The Second Affiliated Hospital, Nanjing Medical University, Jiangsu 210029, China

## Abstract

Type 2 diabetes mellitus (T2DM) and coronary artery disease (CAD) are both characterized by chronic low-grade inflammation. The role of Th17 and its related cytokines in T2DM and CAD is unclear. Here we investigated the serum levels of five Th17-related cytokines (IL-17, IL-22, MIP-3*α*, IL-9, and IL-27) in T2DM, CAD, and T2DM-CAD comorbidity patients. IL-22 was found to be elevated in all three conditions. Elevated serum IL-22 was independently associated with the incidence of T2DM and CAD. Conversely, IL-22 was found to protect endothelial cells from glucose- and lysophosphatidylcholine- (LPC-) induced injury, and IL-22R1 expression on endothelial cells was increased upon treatment with high glucose and LPC. Blocking of IL-22R1 with IL-22R1 antibody diminished the protective role of IL-22. Our results suggest that IL-22 functions as a double-edged sword in T2DM and CAD and that IL-22 may be used in the treatment of chronic inflammatory diseases such as T2DM and CAD.

## 1. Introduction

Type 2 diabetes mellitus (T2DM) and coronary artery disease (CAD) are leading causes of morbidity worldwide. T2DM and CAD are metabolic diseases and share common risk factors such as obesity, hypertension, and dyslipidemia [1]. In addition to the traditional risk factors, chronic low-grade inflammation has now emerged as a major trigger for both these conditions [2]. Accumulating evidences have revealed that multiple types of immune cells are involved in T2DM and CAD pathogenesis [3]. In recent years, the involvement of adaptive immunity in T2DM and CAD has received considerable attention. Activated adaptive immune competent cells are present in atherosclerotic lesions [4, 5]. The presence of activated CD4^+^ T cells in adipose tissue also suggests immune participation in T2DM [6].

As a newly characterized CD4^+^ T-cell subtype, Th17 cells have also been shown to be involved in the pathogenesis of atherosclerosis and T2DM. Th17 cells have been observed to accumulate in murine and human atherosclerotic lesions and have also been suggestive of contributing to inflammation and hyperglycemia in T2DM patients [7–9]. Th17 cells produce a series of cytokines such as IL-17 and IL-22 and are regulated by various cytokines. IL-17, a signature cytokine of Th17, has been reported to be associated with stable plaque phenotype and plaque instability in different trials [10, 11]. IL-22, another cytokine secreted by Th17, was identified to alleviate metabolic disorders in one study. Conversely, in other studies, IL-22 was found to aggravate T2DM condition [12, 13]. MIP-3*α* (also known as CCL20), a ligand for CCR6, plays an important role in the migration of Th17 cells to inflamed sites [14]. IL-9 induces Th17 cells to differentiate and enhances IL-17A production by cultured human peripheral blood mononuclear cells or CD4^+^ T cells [15]. IL-27 induces p-STAT3 to promote Th17 differentiation in the absence of STAT1 [16]. These Th17 regulators also exert their roles in T2DM and CAD, although relevant clinical studies are few [14, 17–19].

In the current study, we evaluated the serum levels of Th17-related cytokines (IL-17, IL-22, MIP-3*α*, IL-9, and IL-27) during immunogenesis. Among these cytokines, IL-22 was found to be elevated in CAD, T2DM, and T2DM-CAD comorbidity patients. In addition, IL-22 was shown to be associated with the incidence of T2DM and CAD. Interestingly, we further established the protective role of IL-22–IL-22R1 in endothelial cells under lysophosphatidylcholine (LPC) and high glucose conditions. Our study indicates that IL-22 might play different roles under different context and suggests that IL-22 may be used for the immunotherapy of chronic inflammatory diseases such as T2DM and CAD.

## 2. Materials and Methods

### 2.1. Study Population

The study was approved by the Institutional Review Board of Nanjing Medical University. Written consent was obtained from each patient. Five-hundred and fifteen patients were recruited from the Second Affiliated Hospital of Nanjing Medical University from 2012 to 2015. Initially, we included 78 people to comprehensively analyze five Th17-related cytokines (Batch 1). Then, 437 people were included for IL-22 analysis (Batch 2). The patients were divided into four groups in each batch: (1) normal healthy volunteers (Batch 1, *n* = 15; Batch 2, *n* = 110); (2) patients with T2DM (Batch 1, *n* = 18; Batch 2, *n* = 58); (3) patients with CAD (Batch 1, *n* = 24; Batch 2, *n* = 132); (4) patients with CAD and T2DM comorbidity (Batch 1, *n* = 21; Batch 2, *n* = 137).

The CAD-group patients were diagnosed on the basis of the results of coronary angiography, and patients with at least one coronary stenosis of >50% of the luminal diameter were included. Furthermore, the stable angina (SA) subgroup included patients with typical effort angina that was accompanied by a downward or horizontal ST-segment depression of >1 mm during an exercise test; the unstable angina (UA) subgroup included patients who exhibited chest pain at rest, with definite ST-segment changes and/or T-wave inversions; the acute myocardial infarction (AMI) subgroup included patients who exhibited significantly elevated levels of creatine kinase MB and cardiac troponin I as well as a typical clinical electrocardiogram manifestation. The CAD group included patients who had normal fasting glucose levels and no history of glucose-lowering medical treatment. The diagnosis of T2DM was based on the results of oral glucose tolerance test (OGTT) and medical treatment history; their coronary angiograms were normal. The control group was recruited according to the following criteria: no history of diabetes, normal glucose tolerance in OGTT, and normal coronary angiograms.

Patients with peripheral vascular disease, thrombotic stroke, nephropathy, retinopathy, inflammatory diseases of any cause, other systemic and metabolic diseases, asthma, malignancy, liver diseases, kidney diseases, and pregnant women were excluded from the study.

### 2.2. Cytokine Quantitative Assays

Serum was obtained by centrifugation and stored at −80°C for the determination of cytokine levels. Protein expression (IL-17, IL-22, MIP-3*α*, IL-9, and IL-27) was measured using a magnetic bead kit based on the Luminex® technology. Briefly, capture antibodies (Millipore) were conjugated to Luminex beads. Detection antibodies (Millipore) were conjugated to biotin through the custom service provided by Millipore. Serum was diluted with an equal volume of MILLIPLEX® MAP cell assay buffer (Millipore). Capture antibody beads were diluted in 25 *μ*L of MILLIPLEX MAP cell assay buffer and added to a magnetic plate (Millipore). Then, 25 *μ*L of the diluted serum was transferred to each well of the solid plate and incubated for 2 h at room temperature with shaking. After incubation, the beads were washed twice with wash buffer, and 25 *μ*L of detection antibodies was added into each well and incubated for 1 h at room temperature with shaking. Then, 25 *μ*L of MILLIPLEX MAP streptavidin-phycoerythrin (Millipore) was added and incubated for 30 min at room temperature with shaking. Finally, sheath fluid was added after washing, and the signal was read using Luminex FLEXMAP 3D*™*.

### 2.3. Serum IL-22 Enzyme-Linked Immunosorbent Assay (ELISA)

Peripheral blood was collected into tubes containing heparin-anticoagulant. Serum was obtained by centrifugation and stored at −80°C for the determination of cytokine levels. Serum IL-22 levels were determined with a quantitative sandwich enzyme immunoassay technique in accordance with the manufacturer's recommendations (Joyee Biotechnics, China).

### 2.4. Cell Culture

Human umbilical vein endothelial cells (HUVECs) were obtained from the American Type Culture Collection (Manassas, VA, USA). HUVECs were cultured in endothelial cell medium (ECM) supplemented with 5% fetal bovine serum, 100 U/mL penicillin, and 0.1 mg/mL streptomycin (all from Sciencell, USA) and incubated at 37°C in 5% CO_2_/95% air environment. When the cells reached 70%–80% confluence, new medium was added before drug treatment.

### 2.5. Cellular Stimuli with a High Concentration of Glucose, LPC, or a Combination of the Two

The original serum and glucose-containing media were removed from the dishes, and the cells were washed three times with phosphate-buffered saline. Various concentrations of glucose (5, 15, 30, 60, and 90 mM) (Sigma, USA) or LPC (5, 10, 20, and 40 *μ*g/mL) (Sigma, USA) were then added to the culture medium for the indicated times (24 or 48 h). To investigate the effects of the combined stimuli, cells were incubated with glucose (30 mM) and LPC (10 *μ*g/mL) for 48 h.

### 2.6. Cell Viability Assays

Cell viability was assessed using CCK-8 according to the manufacturer's recommendations (Kaiji, China). CCK-8 solution (10 *μ*L) was added to each well, and the cultures were incubated at 37°C for 1 h. The results were expressed as the percentage of viable cells relative to the control group after measuring the absorbance at 450 nm. Each experiment included six readings for each experimental condition, and the experiments were repeated thrice.

### 2.7. Apoptosis Assay

Cells were trypsinized with ethylene diamine tetraacetic acid-free trypsin and then washed in serum-containing media. Cells (1 × 10^5^) were collected by centrifugation, resuspended in 100 *μ*L of 1x binding buffer containing 5 *μ*L of Annexin V-FITC and 5 *μ*L of propidium iodide (BD Biosciences, US), and incubated at room temperature in the dark for 15 min. Apoptosis data were acquired on FACSCanto*™* II system (BD Bioscience, US) and were analyzed using the FlowJo software. The results were obtained from three independent experiments.

### 2.8. IL-22R1 Expression Assay

To analyze the expression of IL-22R1, HUVECs were stained with anti-human IL-22R1 (FAB2770P; R&D systems, US). Nonspecific staining was evaluated using phycoerythrin-conjugated mouse IgG as an isotype control (BD Bioscience, US). Data were acquired on FACSCanto II system (BD Bioscience, US) and were analyzed using the FlowJo software. The results were obtained from three independent experiments.

### 2.9. Blockade of IL-22R1 Signaling

To block IL-22R1 in HUVECs, the cells were incubated with human IL-22R1 antibody (AF2770; R&D systems, US) at a concentration of 8 *μ*g/mL for 1 h prior to the addition of IL-22 (PeproTech, US) to the cell culture.

### 2.10. Statistical Analysis

Normally distributed variables were expressed as mean ± standard deviation (SD), nonnormally distributed variables were expressed as median (25th, 75th percentiles), and categorical variables were expressed as numbers (%). For the normally distributed variables, one-way analysis of variance or Dunnett's *t*-test (for comparisons of two groups) was used to compare the cases. For nonnormally distributed variables, the Mann–Whitney *U* test was used to compare the cases, and the Kruskal-Wallis test was used to compare the subgroups. Spearman correlation coefficients were calculated for the associations between the cytokine levels and various laboratory markers. Associations between clinical parameters and the incidence of CAD and T2DM were first analyzed by simple logistic regression analysis and then by multivariate analysis. The odds ratios (ORs) for these variables reflect the change per one unit increase in the measure. *P* < 0.05 was considered to indicate statistical significance. All analyses were performed using SPSS 21.0 for Windows.

## 3. Results

### 3.1. Baseline Characteristics

The clinical characteristics of Batch 1 patients are outlined in Supplementary Material available online at http://dx.doi.org/10.1155/2016/8254797 (TEXT A and Table S1).  Table 1 lists the characteristics of Batch 2 patients. Patients in the T2DM, CAD, and T2DM-CAD groups were older than those in the control group. CAD and T2DM-CAD patients encountered higher incidence of hypertension and stroke than the control individuals. Higher fasting plasma glucose, HbA1c, and urea levels, and lower HDL levels were observed in the T2DM and T2DM-CAD patients relative to the control group.

### 3.2. IL-22 Level Is Increased in T2DM, CAD, and T2DM-CAD Comorbidity Patients

**Figure 1:**
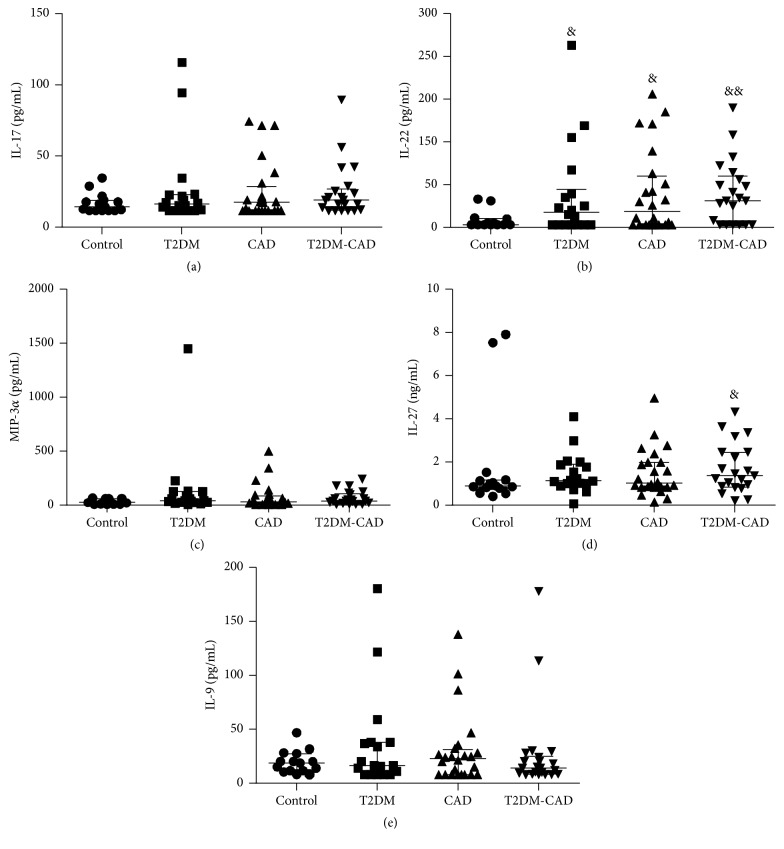
Serum levels of five Th17-related cytokines among the study groups. The serum levels of five Th17-related cytokines were determined in the control, type 2 diabetes mellitus (T2DM), coronary artery disease (CAD), and T2DM-CAD groups by using the Cytokines Quantitative Assays. Values are presented as medians (25th and 75th percentiles). ^&^ *P* < 0.05; ^&&^ *P* < 0.01 versus control group.

In Batch 1 patients, five Th17-related cytokines were analyzed, and significantly higher levels of IL-22 were observed in the T2DM, CAD, and T2DM-CAD comorbidity patients than in the control individuals (Figure 1(b)). However, no differences in IL-17, IL-9, and MIP-3*α* levels were observed across the various groups (Figures 1(a), 1(c), and 1(e)). The IL-27 levels were higher in the T2DM-CAD comorbidity group than in the control group (Figure 1(d)).

**Figure 2:**
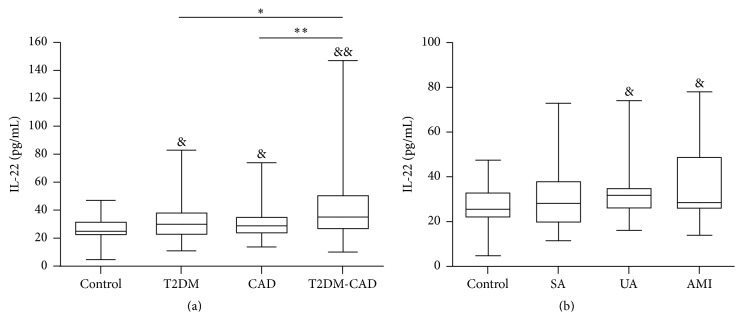
Serum levels of interleukin- (IL-) 22 among the study groups. (a) Serum levels of IL-22 were determined in the control, T2DM, CAD, and T2DM-CAD groups by enzyme-linked immunosorbent assay (ELISA). (b) Serum levels of IL-22 were determined among the CAD subgroups and the control group by ELISA. Values are presented as medians (5th and 95th percentiles). ^&^ *P* < 0.05; ^&&^ *P* < 0.01 versus control group. ^*∗*^ *P* < 0.05, T2DM-CAD versus T2DM group; ^*∗∗*^ *P* < 0.01 T2DM-CAD versus CAD group.

Further expansions of sample size were carried out for a more comprehensive analysis. In Batch 2 analysis, serum IL-22 levels were significantly increased in the T2DM, CAD, and T2DM-CAD comorbidity groups compared with the control group (Figure 2(a)). There was no difference in the IL-22 level between the T2DM and CAD patients. T2DM-CAD patients showed significantly higher levels of IL-22 compared with the T2DM and CAD groups (Figure 2(a)).

Our results also revealed that the IL-22 level was significantly higher in patients with AMI and UA than in the control individuals (patients with AMI, *n* = 27 versus control, *n* = 110, *P* < 0.05; patients with UA, *n* = 53 versus control, *n* = 110, *P* < 0.05; Figure 2(b)). There was no difference in the IL-22 level between patients with SA and healthy individuals (*P* > 0.05).

Table S2 shows Spearman's correlation between IL-22 and the risk factors for T2DM and CAD. IL-22 was negatively correlated with body mass index (*r* = −0.132; *P* = 0.01) and total serum cholesterol levels (*r* = −0.12; *P* = 0.01) and positively correlated with blood urea nitrogen (*r* = 0.105; *P* = 0.04).

Table 2 shows the relationships between various clinical parameters (including IL-22 levels) and the incidence of T2DM, CAD, and T2DM-CAD. Simple logistic regression analysis revealed that age (OR 1.062, 95% CI 1.039 to 1.086; *P* = 0.000000), hypertension (OR 1.750, 95% CI 1.122 to 2.728; *P* = 0.014), stroke (OR 2.978, 95% CI 1.591 to 5.574; *P* = 0.001), high-density lipoprotein (HDL; OR 0.381, 95% CI 0.183 to 0.790; *P* = 0.01), fasting blood sugar (FBS; OR 1.690, 95% CI 1.372 to 2.082; *P* = 0.000001), HbA1c (OR 3.505, 95% CI 2.243 to 5.478; *P* = 0.000000), urea (OR 1.198, 95% CI 1.058 to 1.355, *P* = 0.004), creatinine (OR 1.018, 95% CI 1.007 to 1.028, *P* = 0.001), and IL-22 (OR 1.042, 95% CI 1.023 to 1.062; *P* = 0.000015) were associated with the incidence of CAD and T2DM. The OR values were compared to the control group.

Multiple regression analysis revealed that age (OR 1.082, 95% CI 1.048, 1.111; *P* = 0.000002) and FBS (OR 1.637, 95% CI 1.316 to 2.035; *P* = 0.000009) were significantly associated with the incidence of CAD and T2DM. Notably, IL-22 remained significantly and independently associated with the incidence of CAD and T2DM (OR 1.027, 95% CI 1.004 to 1.050; *P* = 0.022). The OR values were compared to the control group.

### 3.3. LPC and Glucose Decrease the Cell Viability of HUVECs in a Concentration-Dependent Manner

**Figure 3:**
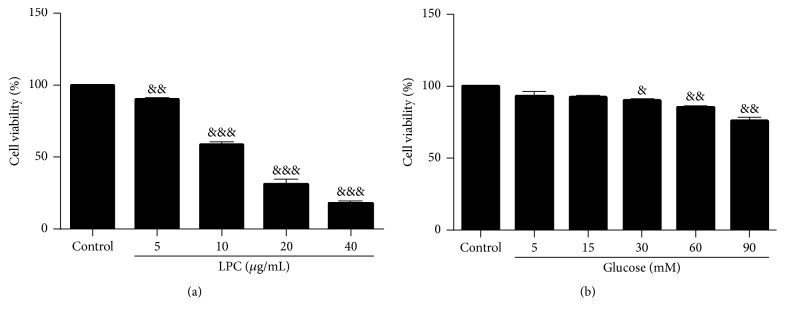
Lysophosphatidylcholine (LPC) and glucose induce human umbilical vein endothelial cell (HUVEC) injury in a concentration-dependent manner. HUVECs were treated with different concentrations of LPC (5, 10, 20, and 40 *μ*g/mL) and glucose (5, 15, 30, 60, and 90 mM) in Dulbecco's Modified Eagle's Medium (DMEM) for 24 h. Cell viability (relative to the control group) was analyzed using the CCK-8 assay. Data shown represent mean ± standard error of the mean (SEM) of three independent experiments. ^&^ *P* < 0.05; ^&&^ *P* < 0.01 versus control group; ^&&&^ *P* < 0.001.

First, to establish a suitable experimental model of HUVEC injury induced by LPC and high concentrations of glucose, we treated HUVECs with different concentrations of LPC (5, 10, 20, and 40 *μ*g/mL) and glucose (5, 15, 30, 60, and 90 mM) in Dulbecco's Modified Eagle's Medium (DMEM) for 24 h and assessed the cell viability by using the CCK-8 assay. As shown in Figure 3(a), the relative optical density (OD) values of cells treated with 5, 10, 20, and 40 *μ*g/mL LPC were 90.25%  ± 5.75%, 58.63%  ± 6.86%, 31.09%  ± 9.07%, and 17.75%  ± 4.88%, respectively. The cell viability of HUVECs treated with 10 *μ*g/mL LPC decreased to 59% relative to that of control group. The relative OD values of cells treated with 5, 15, 30, 60, and 90 mM glucose were 93.11%  ± 7.84%, 92.39%  ± 4.35%, 90.22%  ± 4.64%, 85.38%  ± 4.17%, and 75.9%  ± 10.19%, among which HUVECs treated with 30 mM glucose showed significantly decreased cell viability. These results suggest that 10 *μ*g/mL LPC and 30 mM glucose are the appropriate concentrations to establish HUVEC injury model (Figure 3(b)). We also used 10 *μ*g/mL LPC and 30 mM glucose as the combined stimuli.

### 3.4. IL-22 Protects HUVECs against LPC- and High Glucose-Induced Injury

**Figure 4:**
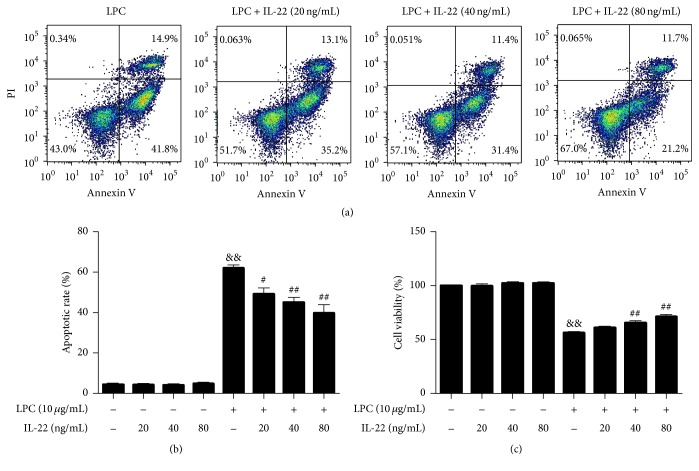
IL-22 protected HUVECs from LPC-induced injury in a concentration-dependent manner. Cells were treated with LPC (10 *μ*g/mL) for 12 h, followed by incubation with different concentrations of IL-22 for 24 h. The apoptotic rate was quantified by flow cytometry. Cell viability was analyzed using the CCK-8 assay and the results were compared with those obtained for the control group. Data shown represent mean ± SEM of three independent experiments. ^&&^ *P* < 0.01 versus control group; ^#^ *P* < 0.05; ^##^ *P* < 0.01 versus LPC-treated group.

**Figure 5:**
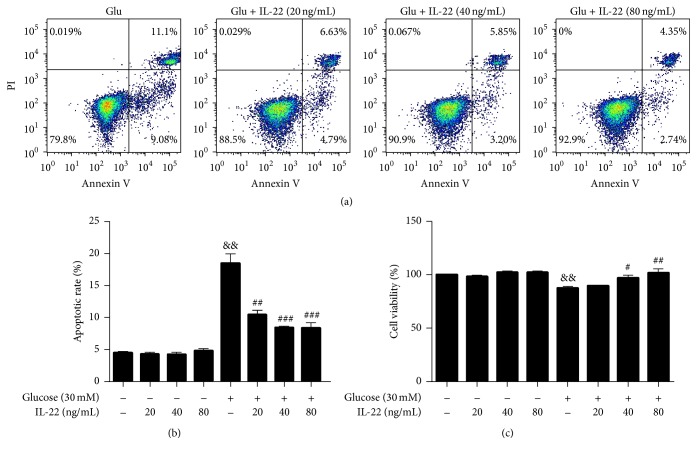
IL-22 protected HUVECs from glucose-induced injury. Cells were treated with glucose (30 mM) for 12 h, followed by incubation with different concentrations of IL-22 for 24 h. The apoptotic rate was quantified by flow cytometry. Cell viability was analyzed using the CCK-8 assay and the results were compared with those obtained for the control group. Data shown represent mean ± SEM of three independent experiments. ^&&^ *P* < 0.01 versus control group; ^#^ *P* < 0.05; ^##^ *P* < 0.01 versus glucose-treated group; ^###^ *P* < 0.0001 versus glucose-treated group.

To determine whether IL-22 has a direct effect on HUVEC cell viability, we treated DMEM cultivated HUVECs with different concentrations of IL-22; however, no change in cell viability was observed in any of the groups (Figure 4(c)). Next, we treated HUVECs with LPC (10 *μ*g/mL) and glucose (30 mM) for 12 h, followed by treatment with different concentrations of IL-22 (20 ng/mL, 40 ng/mL, and 80 ng/mL) for 24 h. Cell viability was assessed by CCK-8 analysis. The cell viability of HUVECs decreased after treatment with 10 *μ*g/mL LPC. In contrast, supplementation with IL-22 abolished the LPC-induced loss of cell viability (Figure 4(c)). The cell viability in the 40 ng/mL IL-22 + LPC group and the 80 ng/mL IL-22 + LPC group was significantly higher than that of the LPC-treated group. These results clearly demonstrated that IL-22 could protect HUVECs from LPC in a concentration-dependent manner. Similarly, in the presence of IL-22 (40 and 80 ng/mL) + 30 mM glucose, the cell viability was higher than that of the glucose-treatment group (Figure 5(c)).

Furthermore, to evaluate the effect of IL-22 on HUVEC apoptosis under different treatment conditions, the cells were incubated with DMEM, LPC, and a high concentration of glucose, accompanied with the addition of different concentrations of IL-22 (20, 40, and 80 ng/mL). No change in apoptotic rate was observed in the DMEM group treated with different concentrations of IL-22 (Figure 4(b)). However, IL-22 effectively attenuated LPC-induced apoptosis in HUVECs in a dose-dependent manner (Figure 4(b)). The percentage of cells undergoing apoptotic cell death decreased from 62.07%  ± 3.9% in the LPC group to 49.2%  ± 8.6%, 45.18%  ± 8.5%, and 39.83%  ± 10.1% after being exposed to 20, 40, and 80 ng/mL IL-22, respectively, for 24 h. Meanwhile, the apoptotic cell death decreased from 18.47%  ± 2.8% in the high glucose group to 10.4%  ± 1.5%, 8.4%  ± 0.3%, and 8.33%  ± 1.7% after being exposed to 20, 40, and 80 ng/mL IL-22, respectively, for 24 h (Figure 5(b)).

### 3.5. IL-22R1 Expression on HUVECs Was Enhanced upon Stimulation with LPC and High Concentrations of Glucose

**Figure 6:**
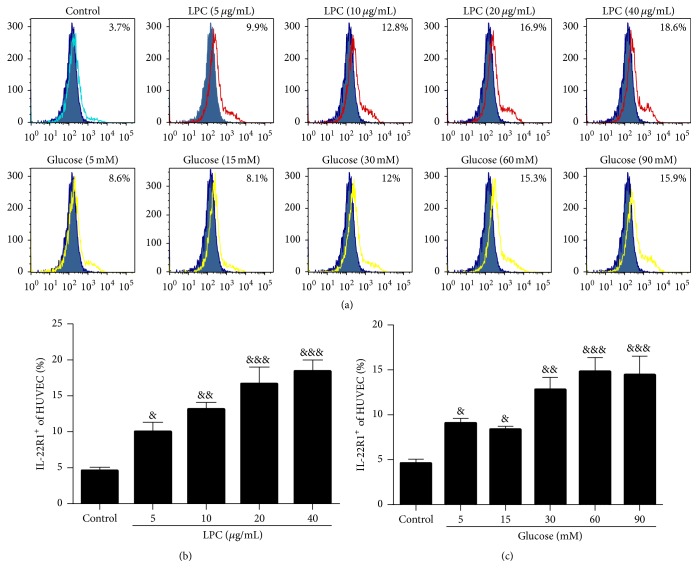
LPC and high concentrations of glucose increased IL-22R1 expression in HUVECs. Representative flow cytometry histogram of IL-22R1 expression on HUVECs. Surface expression of IL-22R1 (open area) on HUVEC single-cell suspensions in comparison with the isotype control (filled area), as determined by flow cytometry. Green open area represents control group. Red open area represents LPC group. Yellow open area represents glucose group. The percentages of IL-22R1^+^ cells among the HUVECs were analyzed by using FlowJo. Data shown represent mean ± SEM of three independent experiments. ^&^ *P* < 0.05, ^&&^ *P* < 0.01, and ^&&&^ *P* < 0.0001 versus control group.

The IL-22 receptor is a heterodimer composed of two subunits, IL-22R1 and IL-10R2. We attempted to detect IL-22R1 expression on HUVECs after treatment with high concentrations of glucose and LPC by flow cytometry. As shown in Figure 6(b), HUVECs exposed to LPC (10, 20, and 40 *μ*g/mL) for 24 h exhibited a dose-dependent increase in IL-22R1 expression (13.13%  ± 2.77%, 16.68%  ± 6.15%, and 18.45%  ± 4.19%, resp.) compared with the DMEM-treated group (4.87%  ± 1.57%). HUVECs treated with high concentrations of glucose (30, 60, and 90 mM) also exhibited increased IL-22R1 expression (12.83%  ± 2.97%, 14.83%  ± 3.67%, and 14.45%  ± 4.41%, resp.) compared with the DMEM-treated group (Figure 6(c)).

### 3.6. Blocking IL-22R1 Eliminated the Protective Role of IL-22 against LPC-, High Glucose-, and Combined Stimuli-Induced Injury on HUVECs

**Figure 7:**
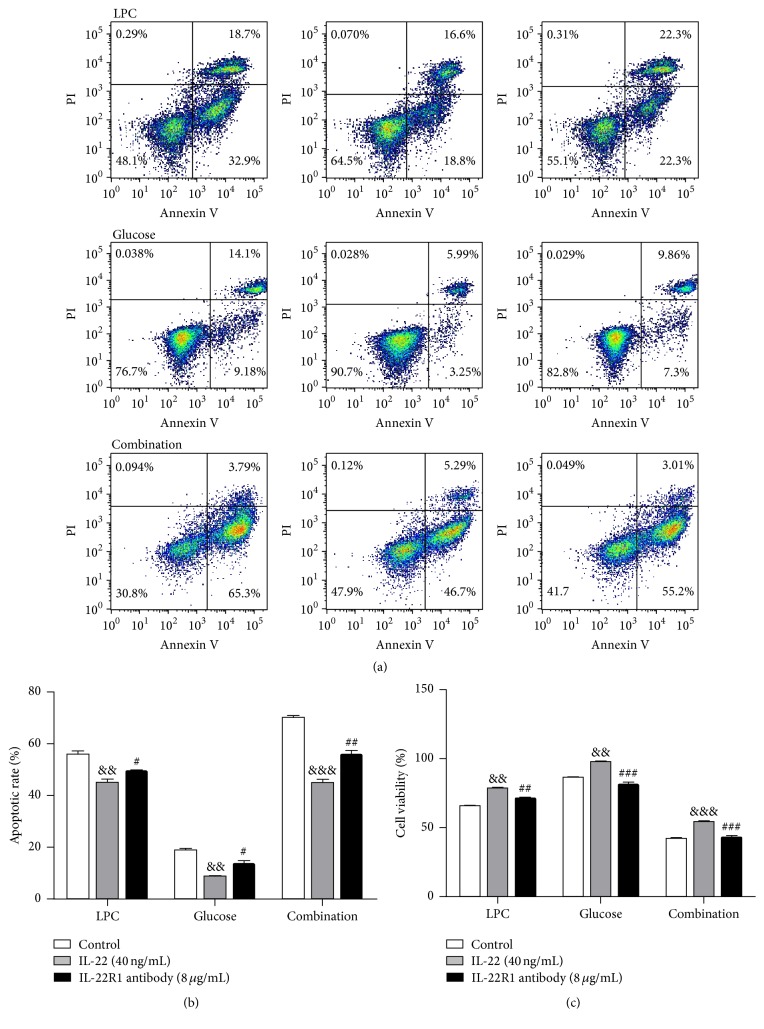
Treatment with IL-22R1 antibody ameliorated the protective role of IL-22 in LPC-, glucose-, or combination-treated HUVECs. Cells were treated with LPC (10 *μ*g/mL), glucose (30 mM), or a combination of the two (10 *μ*g/mL LPC + 30 mM glucose) for 12 h followed by incubation with 40 ng/mL IL-22 or 0.8 *μ*g/mL IL-22R1 antibody + 40 ng/mL IL-22 for 24 h. IL-22R1 antibody (8 *μ*g/mL) was added 1 h before treatment with 40 ng/mL IL-22. The apoptotic rate was quantified by flow cytometry. Data shown represent mean ± SEM of three independent experiments. ^&&^ *P* < 0.01 versus control group; ^&&&^ *P* < 0.0001 versus control group. ^#^ *P* < 0.05 versus IL-22-treated group; ^##^ *P* < 0.01 versus IL-22 treated group; ^###^ *P* < 0.0001 versus IL-22 treated group.

Because IL-22-targeting cells are restricted to nonimmune cells that express IL-22R1, we hypothesized that IL-22 exerts its protective role via IL-22R1. Therefore, we carried out IL-22R1 blocking experiments. IL-22 (40 ng/mL) increased the relative cell viability of LPC-, high glucose-, and LPC + high glucose-treated HUVECs from 64.88%  ± 3.42%, 86.09%  ± 2.85%, and 41.7%  ± 4.66% to 78.14%  ± 3.23%, 97.21%  ± 4.3%, and 53.83%  ± 5.21%, respectively. However, in the IL-22 + IL-22R1 antibody-treated group, the cell viability of HUVECs decreased to 70.68%  ± 2.67%, 78.46%  ± 8.57%, and 42.6%  ± 5.73%, respectively (Figure 7(c)).

In addition, 40 ng/mL IL-22 decreased the apoptotic rate of LPC-, high glucose-, and LPC + high glucose-treated HUVECs from 56.37%  ± 1.44%, 18.47%  ± 2.84%, and 69.9%  ± 2.85% to 44.5%  ± 4.71%, 8.4%  ± 0.26%, and 44.87%  ± 3.32%, respectively. However, in the IL-22 + IL-22R1 antibody-treated group, the apoptotic rate of HUVECs increased to 49.27%  ± 1.05%, 13.37%  ± 2.21%, and 55.6%  ± 1.73%, respectively (Figure 7(b)).

## 4. Discussion

Our results clearly demonstrated that patients with T2DM and CAD exhibit increased serum IL-22 levels. Elevated serum IL-22 is associated with the incidence of CAD and T2DM. However, further* in vitro* study established the protective role of IL-22 against endothelial dysfunction, an essential process involved in the early development of atherosclerosis and vascular complications in T2DM [20, 21]. Potential stimuli present in T2DM and CAD condition, such as high glucose and LPC, lead to increased IL-22R1 expression on endothelial cells. To the best of our knowledge, this is the first report on the effect of these stimuli on IL-22R1 expression. Neutralizing IL-22R1 with IL-22R1 antibody diminished the protective role of IL-22. Together, our results suggest that IL-22 might exert different functions under different context. When used appropriately, IL-22 may have applications in the treatment of chronic inflammatory diseases such as T2DM and CAD.

In our study as well as in some previous studies, IL-22 was found to be increased in UA, AMI, and T2DM patients. However, no further mechanism-related research was reported before [22–24]. Our clinical study indicated the pathologic effects of IL-22. Our* in vitro* study, however, established the protective role of IL-22 in the endothelium. The beneficial effect of IL-22 reported here is consistent with some previous findings regarding its role in diabetes and atherosclerosis. Islet-endogenous and islet-exogenous IL-22 are able to suppress oxidative and endoplasmic reticulum stress caused by cytokines or glucolipotoxicity in mouse and human beta cells [25]. Additionally, IL-22 treatment improves keratinocyte prohealing functions in diabetes patients, suggesting a therapeutic potential for IL-22 in diabetic ulcer management [26]. In the case of atherosclerosis,* IL-22* ^−/−^ *Apoe* ^−/−^ mice exhibited reduced plaque size and decreased plaque collagen level, which are important in the maintenance of plaque stability. IL-22 might promote plaque growth and contribute to plaque stability during lesion formation [27].

In this study, we discovered an interesting “paradox.” Our clinical study revealed that elevated serum IL-22 is correlated with the incidence of T2DM and CAD independent of other clinical parameters. However, our cellular study showed that IL-22 might contribute to endothelium regeneration. The results are not mutually exclusive. As reported by Dudakov et al., upon acute tissue damage (such as in the case of our cellular model), IL-22 is presumably able to migrate to local lesions to promote endothelium survival and proliferation. However, long periods of chronic IL-22 overexpression (such as in our clinical context) could lead to the production of chemokines and other inflammatory signals and subsequent recruitment of pathologic effector cells to the inflamed tissues, leading to deterioration of disease condition [28]. In chronic hepatitis, IL-22-related pathology is dependent on the recruitment of Th17 cells [29]. The proinflammatory role of IL-22 has also been reported in the case of T2DM [12]. IL-22 amplifies IL-1*β* driven inflammation in human adipose tissue, thereby disrupting glucose homeostasis in obesity and T2DM. Whether IL-22 mediates a physiologic response or contributes to pathophysiologic inflammation probably depends on the context and/or cytokine milieu.

IL-22R is composed of two heterodimeric subunits, IL-22R1 and IL-10R2 [28]. IL-22 has a high affinity for IL-22R1 but no affinity for IL-10R2. Initial binding of IL-22 to the IL-22R1 subunit enables secondary binding of the IL-10R2 subunit, thereby enabling downstream signaling. The IL-22–IL-22R1 pathway has been reported to be able to contribute to migration and proliferation in hyperglycemic keratinocytes both* in vitro* and* in vivo* by inducing STAT3 phosphorylation [26]. Activation of the keratinocyte proproliferative and prosurvival ERK1/2 pathways by IL-22 has also been observed in both hyperglycemic and diabetic patients [30]. Therefore, the IL-22–IL-22R1 signaling pathway in our context warrants further study.

Targeting the IL-22–IL-22R1 system might benefit chronic inflammatory diseases concerning the skin, chronic infections, pancreatitis, and so forth, although therapeutic modulation should be individualized [31]. In the case of psoriasis, inhibition of IL-22–IL-22R1 is recommended because the persistent regenerative role of IL-22 is pathogenetic [32]. On the other hand, pancreatitis might benefit from supplementation of IL-22 because of its protective effects on pancreatic acinar cells and islet cells [33, 34]. Thus, the IL-22–IL-22R1 system can be protective or pathological depending on the context. Concerning that IL-22 might function like a double-edged sword during T2DM and CAD pathogenesis, the timing of IL-22–IL-22R1 application is crucial during the treatment of T2DM and CAD. Appropriate enhancement or reduction of IL-22–IL-22R1 depending on the context might have the potential to treat chronic inflammatory diseases such as T2DM and CAD in a comprehensive manner.

There are some limitations in our study. Because IL-22 is generated from a variety of T-cell subsets such as Th17, Th22, and *γδ* T cells, further analysis is required to track the origin of IL-22 and explore the function of the concerned immune cells. In addition, cross-sectional studies have intrinsic limitations. Whether the expression of IL-22 has a dynamic effect needs further investigation. In-depth analysis is underway to determine whether IL-22 is able to translate into a therapy for T2DM and CAD.

## 5. Conclusions

In conclusion, our results demonstrated that the serum IL-22 level is higher in T2DM, CAD, and T2DM-CAD comorbidity patients than in controls. Elevated serum IL-22 is associated with the incidence of T2DM and CAD. IL-22 is able to protect endothelial cells against LPC- and high glucose-induced injury. IL-22R1 might contribute to this beneficial effect of IL-22. Our results imply that IL-22 is a double-edged sword in CAD and T2DM. When used appropriately, IL-22 may have applications in the treatment of chronic inflammatory diseases such as T2DM and CAD.

## Supplementary Material

The supplementary material include Text A, Table S1, and Table S2. Text 1 describes the clinical and biochemical characteristics of Batch 1 patients. Table S1 shows the clinical characteristics of the Batch 1 study individuals among different groups. Table S2 shows the Spearman rank correlations between IL-22 and the established laboratory markers for T2DM and CAD.

## Figures and Tables

**Table 1:** Clinical characteristics of Batch 2 patients.

Clinical parameters	Control (*n* = 110)	T2DM (*n* = 58)	CAD (*n* = 132)	T2DM-CAD (*n* = 137)
Age, years	58.15 ± 9.84	61.16 ± 14.38^*∗*^	65.67 ± 9.93^*∗∗∗*^	65.69 ± 9.82^*∗∗∗*,&^
Men, *n* (%)	54 (49.1)	35 (60.3)	79 (59.8)	67 (48.9)
BMI (kg/m^2^)	24.66 (22.86, 25.38)	23.86 (22.26, 26.99)	24.33 (22.6, 25.36)	24.72 (22.97, 26.12)
Hypertension, *n* (%)	62 (56.4)	30 (51.7)	92 (69.7)^*∗*^	103 (75.2)^*∗∗*,&&^
Stroke, *n* (%)	13 (11.8)	13 (22.4)	32 (24.2)^*∗*^	48 (35)^*∗∗∗*,#^
Smoke, *n* (%)	34 (30.9)	10 (17.2)^*∗*^	51 (38.6)^*∗*^	46 (33.6)^&^
Alcohol consumption, *n* (%)	13 (11.8)	5 (8.6)	26 (19.7)	16 (11.7)
FBS (mmol/L)	5.2 (4.91, 5.53)	6.39 (7.65, 11.07)^*∗∗∗*^	5.36 (4.88, 5.84)	7.83 (6.53, 10.32)^*∗∗∗*,###^
HbA1c (%)	4.9 (4.78, 5.2)	6.4 (5.55, 8.25)^*∗∗∗*^	5.1 (4.7, 5.5)	6.5 (5.6, 7.8)^*∗∗∗*,###^
TC (mmol/L)	4.48 ± 0.3	4.56 ± 1.11	4.54 ± 1.02	4.56 ± 1.26
TG (mmol/L)	1.11 (0.77, 1.76)	1.39 (0.84, 1.89)	1.29 (0.91, 1.86)	1.42 (1.11, 2.26)^*∗*^
HDL (mmol/L)	1.25 ± 0.31	1.11 ± 0.37^*∗*^	1.21 ± 0.33	1.09 ± 0.32^*∗∗*,#^
LDL (mmol/L)	2.54 ± 0.77	2.48 ± 0.92	2.62 ± 0.76	2.57 ± 1.01
Urea (mmol/L)	5.38 (4.65, 6.43)	6.07 (4.76, 7.26)^*∗*^	5.58 (4.54, 6.85)	6.19 (5.02, 7.83)^*∗∗*^
Creatinine (mmol/L)	66.2 (55.9, 80.35)	74.3 (61.1, 90.2)^*∗*^	77.65 (63.68, 93.63)^*∗∗∗*^	75 (58.05, 88.05)

BMI, body mass index; CAD, coronary artery disease; FBS, fasting blood sugar; HbA1c, hemoglobin A1C; HDL, high-density lipoprotein; LDL, low-density lipoprotein; T2DM, type 2 diabetes mellitus; TC, total cholesterol; TG, triglycerides. Data are presented as mean ± standard deviation or median (25th percentile; 75th percentile).

Alcohol consumption refers to an average ethanol consumption up to 15 g/day for women and 30 g/day for men.

^*∗*^ *P* < 0.05, ^*∗∗*^ *P* < 0.01, and ^*∗∗∗*^ *P* < 0.0001 versus control group.

^&^ *P* < 0.05 and ^&&^ *P* < 0.01 versus T2DM group.

^#^ *P* < 0.05 and ^###^ *P* < 0.0001 versus CAD group.

**Table 2:** Logistic regression analysis for the incidence of T2DM, CAD, and T2DM-CAD.

Clinical parameters	Simple regression		Multiple regression	
	OR (95% CI)	*P*	OR (95% CI)	*P*
IL-22 (pg/mL)	1.042 (1.023, 1.062)	0.000015	1.027 (1.004, 1.050)	0.022
Age (per/yr)	1.062 (1.039, 1.086)	0.000000	1.082 (1.048, 1.111)	0.000002
Sex (male)	1.295 (0.840, 1.996)	0.243		
BMI (kg/m^2^)	0.991 (0.920, 1.067)	0.811		
Hypertension (yes)	1.750 (1.122, 2.728)	0.014	0.916 (0.418, 2.009)	0.827
Stroke (yes)	2.978 (1.591, 5.574)	0.001	0.940 (0.338, 2.614)	0.905
Smoking (yes)	0.937 (0.587, 1.494)	0.783		
Drinking (yes)	1.121 (0.613, 2.051)	0.712		
TC (per mmol/L)	1.062 (0.851, 1.326)	0.595		
TG (per mmol/L)	1.210 (0.946, 1.548)	0.129		
HDL (per mmol/L)	0.381 (0.183, 0.790)	0.01	0.433 (0.152, 1,227)	0.115
LDL (per mmol/L)	1.043 (0.789, 1.379)	0.766		
FBS (per mmol/L)	1.690 (1.372, 2.082)	0.000001	1.637 (1.316, 2.035)	0.000009
HbA1c (%)	3.505 (2.243, 5.478)	0.000000	1.388 (0.636, 3.031)	0.411
Urea (per mmol/L)	1.198 (1.058, 1.355)	0.004	0.905 (0.747, 1.096)	0.307
Creatinine (per mmol/L)	1.018 (1.007, 1.028)	0.001	1.007 (0.988, 1.026)	0.411

## Abbreviations

CAD: Coronary artery disease
LPC: Lysophosphatidylcholine
T2DM: Type 2 diabetes mellitus.
