# Supplementary material for: Interleukin-22 Might Act as a Double-Edged Sword in Type 2 Diabetes and Coronary Artery Disease
Source: Mediators Inflamm. 2016 Oct 18;2016:8254797. doi: 10.1155/2016/8254797 (PMC5088317; doi:10.1155/2016/8254797)
Supplement: Supplementary file 1 — The supplementary material include Text A, Table S1, and Table S2. Text 1 describes the clinical and biochemical characteristics of Batch 1 patients. Table S1 shows the clinical characteristics of the Batch 1 study individuals among different groups. Table S2 shows the Spearman rank correlations between IL-22 and the established laboratory markers for T2DM and CAD. [file 8254797.f1.pdf]

TEXT A

Baseline Characteristics

Table S1 shows the clinical and biochemical characteristics of Batch 1 patients. Compared to the control group, T2DM patients were older, had a higher BMI, were more likely to smoke, and had a higher incidence of stroke. Concerning the laboratory markers, T2DM patients showed higher fasting plasma glucose, HbA1c, and urea values. CAD patients were significantly older and more likely to have hypertension than the control individuals. Among the laboratory markers, CAD and T2DM-CAD co-morbidity patients had higher levels of creatinine compared to the control individuals; 61.9 % of the T2DM-CAD patients had hypertension. Fasting plasma glucose, HbA1c, and creatinine values were also higher in the T2DM-CAD patients than in the control group.

Table S1. Clinical characteristics of the Batch 1 study individuals.

**Clinical parameters**	**Controls (n = 15)**	**T2DM (n = 18)**	**CAD (n = 24)**	**T2DM-CAD (n = 21)**
Age, years	52 ± 9.5	62 ± 9.7**	64.5 ± 8.9***	64 ± 11.1**
Men, n (%)	5/15	7/18	20/24	8/21
BMI (kg/m^2)	22.7 ± 1.8	25.2 ± 4.5*	23.1 ± 1.8	24 ± 2.8
Hypertension, n (%)	2/15	10/18	14/24**	13/21**
FBS (mmol/L)	5.04 ± 0.31	7.40 ± 0.78*	5.13 ± 0.78	9.04 ± 2.88***
HbA1c (%)	4.73 ± 0.42	6.17 ± 1.18**	4.91 ± 0.47	7.83 ± 1.73***
TC (mmol/L)	4.66 ± 1.00	4.28 ± 1.01	4.61 ± 1.00	5.09 ± 1.6
TG (mmol/L)	0.95 (0.74, 1.76)	1.36 (0.67, 2.21)	1.09 (0.91, 1.38)	1.3 (1.10, 2.5)
HDL (mmol/L)	1.37 ± 0.35	1.14 ± 0.39	1.25 ± 0.33	1.29 ± 0.31
LDL (mmol/L)	2.71 ± 0.87	2.27 ± 0.97	2.74 ± 0.81	12.64 ± 0.98
Urea (mmol/L)	5.46 ± 1.65	6.95 ± 2.7*	5.84 ± 2.21	6.33 ± 1.79
Creatinine (mmol/L)	61.48 ± 12.44	74.42 ± 26.01	86.95 ± 28.21**	79.59 ± 29.84*

Data are presented as mean ± standard deviation or median (25th percentile, 75th percentile)

$^{*}P < 0.05$, $^{**}P < 0.01$, $^{***}P < 0.0001$ vs. control group.

Table S2. Spearman rank correlations between IL-22 and the established laboratory markers for T2DM and CAD.

IL-22	*R*	*P*
BMI	−0.132	0.01**
TC	−0.12	0.01**
TG	−0.059	0.286
HDL	−0.72	0.322
LDL	−0.072	0.2
BUN	0.105	0.04*
FBS	0.047	0.407
HbA1c	0.055	0.333
